# Book Reviews

**Published:** 1820-08

**Authors:** 


### CRITICAL ANALYSIS

OF

### RECENT PUBLICATIONS, IN THE DIFFERENT BRANCHES OF MEDICINE AND SURGERY.

"I would have men know, that though I reprehend the easie passing over of the causes of things, "by ascribing them to secret and hidden vertues and properties; (for this hath arrested and laid "asleepe all true enquiry and indications;) yet I doe not understand but that, in the practical "part of knowledge, much will be left to experience and probation, whereunto indication cannot "so fully reach: and this not only in *specie*, but in *individuo*. Yet it was well said, *Vere scire* "*esse per causas scire*."—BACON.

***A History of the Epidemic Fever which prevailed in Bristol during the Years* 1817, 1818, and 1819; *founded on Reports of St. Peter's Hospital and the Bristol Infirmary.*** By J. C. PRICHARD, M.D. late of Trinity College, Oxford; Fellow of the Linnæan and Wernerian Societies, &c. Physician to St. Peter's Hospital and the Bristol Infirmary. 8vo. pp. 112. J. and A. Arch, London. 1820.

NOTWITHSTANDING the numerous works on Fever, in professed Treatises and Hospital Reports, which have been recently produced, writings of this kind still elicit our attention with almost as much earnestness as though the discussion of the subject were still new; for we have yet much to learn respecting the nature of this form of disease, that relates in an intimate manner to several very important indications for its medical treatment. It is become almost universally acknowledged, that topical inflammation, or local disease presenting the sensible characteristics of inflammation, always accompanies fever; but it is still disputed whether this local inflammation is a consequence of the more general disturbance of the system, an accidental contingency, or, as DARWIN's theory indicates, the primary and essential cause of its development. Some who adopt the last opinion argue, further, that it is essential that this inflammation should exist in a certain organ, as the brain; whilst others believe that the mucous membrane must be the seat of it. BROUSSAIS has nearly made up his opinion, that irritation of the mucous membrane of the alimentary canal is the essential cause of the symptoms which are generally considered to characterize fever; a notion which originated with Dr. EDWARD MILLER, of New-York, and which is exposed in that excellent physician's Dissertation on the Sympathy of the Stomach. The truth of this doctrine, as far as regards *eruptive fevers*, has, we consider, been rendered very probable, if not demonstrated, by Dr. RICHARD HARRISON, in the paper inserted in the last number of this Journal.

No one can deny that the discovery of the truth is not here a very desirable object; for, though the doctrine that fever is closely allied with an inflammatory diathesis, has led to the general use of the most heroic and efficacious remedies, more accurate knowledge of the relations above designated must enable us to apply them with greater precision, and probably teach us how to employ several beneficial auxiliaries with greater effect than we can do at present, with our indistinct views of the order and relations of the phenomena of the malady.

The object of the work before us is to furnish a report of the author's observations and the mode of practice adopted in St. Peter's Hospital and the Infirmary at Bristol; and therefore does not profess to give any thing like a general theory of fever. It is, however, possible to arrange observations, with the immediate inferences from them, in such a manner, as to present views of the subject that have almost the character of a code of doctrine; of which we have specimens in the Reports of the Edinburgh Infirmary, by Dr. DUNCAN, and in those of the Fever Hospitals of Dublin and Cork, and which Dr. Prichard professes to have taken as his models. These reports have been the means of elucidating many of the most interesting points in the history of fever, and of diffusing amongst the profession generally the best precepts for its treatment. Though the production before us does not present any thing of remarkable novelty in those respects, it furnishes testimony of such a kind in favour of what had been previously proposed, as renders it a highly valuable addition to the records above mentioned. There is a clearness and precision, with a strength of manner in demonstrating facts and indicating relations, in this work, that give extraordinary force to what the author desires to fix in the mind of the reader; and, as the doctrines it inculcates are consonant with those supported by the best authorities, it must be regarded as an addition to medical literature of considerable importance; as a work conducive to the improvement of the practice of medicine, as well as a record in the history of the fever which has prevailed so extensively throughout Great Britain in the last memorable decade.

Productions of this kind are not adapted for particular criticism: there are no hypotheses or theories to examine, and but few peculiar opinions to discuss the propriety of: the only question in this respect is, whether or not the author has effected his object in a worthy manner. On this point we have already given our judgment. Neither are they proper for analytical abridgment, when already drawn up in an abstract form, comprising only matters of essential importance, like that before us. Still, in conformity with our usual practice, we shall endeavour to give such a view of the striking traits of it, as will enable the reader to form a tolerably accurate idea of its general character. We commence with some preliminaries calculated to explain several circumstances of importance, in the allusions to the history of the epidemic fever of Bristol, that will hereafter occur.

The city of Bristol had been for several years remarkably free from fever, until 1817; since when it has participated with the other towns of Great Britain in the visitation of this malady. The public institutions for the reception of the patients of it in Bristol are, St. Peter's Hospital and the Infirmary. The former is not a building erected for the accommodation of the sick: it is the general poor-house of the whole city, and formed of a confused heap of buildings appended one to the other, each part having been raised to answer some occasional purpose; and, consequently, there are many parts of the hospital which it is impossible to ventilate, or to render decent and comfortable. The inhabitants are, on an average, about 420; a considerable proportion of them being changed repeatedly in the course of a year. They consist of three classes of persons: the first, and most numerous, are of the same description as the inmates of poor-houses in general; the second includes all the vagrants and beggars who are found in Bristol; they do not remain long in the hospital, but are sent forwards to their respective homes as speedily as possible, and consequently there is a frequent succession of them. The third are the idiots and lunatics of the lower orders, from the city, and the towns and villages in its vicinity.

The number of cases of fever admitted into this hospital during the last seven months of 1817, was 95; that in 1818, 150; and that in 1819, 105; a concise account of which, individually, is presented by the author, in the form of tables. Of the 95 cases which occurred in 1817 there were twenty fatal; of the 150 in 1818, sixteen; and only eleven in the 105 cases in the last year. The large proportion of deaths is satisfactorily accounted for, from the circumstances under which the individuals were commonly admitted into the hospital. Many were brought in from the streets in a hopeless condition; some of them, subsequently to the attack of fever, had been lying in lanes or on the road-sides, without shelter from the inclemencies of the weather, and destitute of the means for supporting life under ordinary circumstances; others had lain in confined hovels, and without any medical attendance, until the time for relieving them was past. Besides these circumstances, it was the general practice to introduce the worst cases that occurred amongst the out-patients into the house, in order to have a better opportunity of attempting their relief; and therefore the list of in-patients may be considered as a selection of the severest examples of disease out of the whole number of cases.

Thus, the whole number of deaths, of both in and out patients, during the period above mentioned, amounted only to 54, though the total number of cases was 591.

The number of patients admitted into the Infirmary during the same period, was 417. That for the two latter years alone, in which the number of deaths was noticed, was 370, and the fatal cases in these 24; 16 of which occurred in 1818 amongst 172 patients, and eight in 1819 amongst 198. It is here necessary to remark, that the cases in the Infirmary were selected from the out-patients, all those presenting alarming symptoms being admitted; and therefore the number of deaths should be properly calculated in relation to the aggregate of the whole, as it is probable that fatal cases were confined to the hospital. By this mode of calculation, there were only 24 deaths in 575 cases. In the year 1818, too, many distressed vagrants were admitted, who were brought in in a state of extreme exhaustion; which will explain why the relative mortality was much greater in that than in the last year, when such patients were not equally numerous.

After the preliminary details from which we have formed the above abstract, the author makes some general "observations on the Symptoms, Pathology, and Medical Treatment, of Fever." His observations on the first two points are drawn up with sufficient conciseness to permit us to transcribe them without abridgment.

"That the derangement of the system of functions which constitutes fever, is very nearly allied in its nature to the disease which accompanies the inflammation of particular organs, is an opinion which is gaining ground every day among medical practitioners. It is, indeed, very remarkable that they have been so long in arriving at it, when we consider the striking analogy in the phenomena of these distempers. Fever of almost every type and variety is occasionally imitated so closely by morbid states that occur in the phlegmasiæ, that it is sometimes necessary to make a diligent enquiry into circumstances, in order to distinguish them. It was long ago remarked by an eminent practical writer, that there is no one pathognomic symptom by which fever, properly so called, may be discriminated.*

Whether fever and inflammatory disease are identical, in respect to the disordered action which constitutes them, will be rather a matter of curiosity than of useful enquiry, so long as we remain ignorant in what inflammation really consists. While one party of physiologists declare that inflammation is increased action of the arteries, and another that it is diminished action, while a third avers that the arteries are never capable of any action at all, we cannot hope to throw much light on the nature of fever, even if we could prove that fever is inflammation. But the fact that these diseases are nearly allied, and that the former is very

* Dr. G. Fordyce's Essays on Fever.

often productive of the latter, is of great practical importance, since we happen to understand more fully than most other parts of medical practice, how to treat inflammatory disorders. And the fact I have just alluded to must be allowed without controversy by every person who is possessed of competent knowledge. Fever is only dangerous when it gives rise to, or displays, the symptoms of visceral inflammation.

"In that milder form of the disease which is termed simple fever, there is no certain proof of any local inflammation. The blood, however, when drawn, even under these circumstances, often exhibits, as it was observed by Sydenham, the inflammatory crust; the state of the secretions is similar to the condition they assume in the phlegmasiæ; the relief also which is produced by venesection, and by other evacuations, tends to prove that the disease is in its nature analogous to these distempers. But this milder species is ever liable to be converted into a severer fever, exhibiting both in the living and the dead body unequivocal marks of inflammation in the brain, the lungs, the liver, stomach, or bowels. Simple fever most naturally and frequently degenerates into cephalic: indeed, the cases to which these names are respectively applied, are only distinguished by the different violence in the symptoms; the head is more or less affected with pains and other disorders, in both cases. The seat of these, in the more severe form, seem to be the membranes of the brain; and to the same parts, as well as to the coverings of the nerves, we may, with great probability, ascribe the pains in the head and limbs which attend the first attack of almost every case of fever.

"Simple and cephalic fever may therefore be considered as the genuine forms of this disease, the attack being milder in the first, and more severe in the second. The pneumonic, hepatic, gastric, enteric, and rheumatic, forms may be regarded as varieties."

We shall not stop to repeat the opinions on this subject we have so often expressed in the late numbers of this Journal; but proceed to the consideration of the mode of treatment pursued by the author, which is remarkable in several respects, and especially in the extent to which he employed blood-letting in patients previously half-exhausted by famine and fatigue, and in the advanced stages of the disease. The author, in these respects, has surpassed the warmest of former advocates for blood-letting. The decisive advantages obtained from the measure are, of course, the best arguments for the use of it; and one remark alone is sufficient to oppose the suppositions of those who imagine that the advantages obtained from it in some cases may have been counterbalanced in others by injurious consequences: in the more severe forms of cephalic fever, (in which bleeding was most extensively employed,) when the disease was not subdued by the means resorted to, "it was almost uniformly proved, by examination after death, that the brain had suffered irreparable injury."

Dr. Prichard, we should have before stated, designates fever according to the organs which develop the most striking symptoms, as cephalic, &c. ; and he calls simple fever, that form in which there are not clearly manifest signs of inflammation of any certain organ. The propriety of this is indisputable in a Report like that before us. But with this acknowledgment we must join the remark, that the predominance of cephalic or pulmonary symptoms, and the absence of striking signs of gastric irritation, do not show, as some urge, that gastric irritation is not present, and has not been the primary link in the chain of the proper febrile phenomena. Every person of extensive research in pathological anatomy, must have had occasion to witness proofs of the fact mentioned by CULLEN, (*First Lines*, vol. i. p. 205,) that inflammation of the stomach may exist, and prove fatal, without being accompanied with pain or vomiting. A case is related in Dr. DUNCAN's *Medical Commentaries*, (vol. iii. 2d decade, p. 146,) of cancerous affection of the stomach, the existence of which had never been indicated by nausea or retching. And, what is still more interesting in regard to fever, and especially to *eruptive fevers*, MORGAGNI states a case (*De Sedibus*, vol. iii. p. 374), of a dose of arsenic proving fatal, where no inflammation, erosion, or sign of injury, of the stomach appeared after death ; yet the mineral was found in contact with the mucous membrane of that organ, was detected by the usual chemical tests, and had produced one of its common effects when thus administered—*livid spots on the skin.*

In a considerable proportion of the patients brought to St. Peter's Hospital, and in many of those who were admitted into the Infirmary, the disease was still in its first stage, or in the period of depressed vital action. "The cold stage," the author continues to observe, "often seemed to have been protracted beyond its usual duration in vagrants and other paupers, in consequence of exposure to cold and damp winds, by lying during wet nights on the sides of roads, or in other unsheltered places ; and by the want of all sustenance and means of restoring warmth and the vigour of the circulation." In some this state continued for several days, the patients having "never experienced the hot fit, or the return of arterial energy ; their pulse was feeble and depressed, their extremities cold, and their skin clammy." Occasionally they sunk, in spite of all efforts to restore warmth and animation : in other instances, "re-action, when it at length took place, was extremely violent, and produced a high degree of inflammation in some of the viscera, which, occurring as it did, in habits previously exhausted and unable to sustain its violence, speedily terminated in effusion, suppuration, or gangrene, and in death."

The means first employed with such patients were those which

Dr. JACKSON, and the authors of the Reports of the Dublin Fever Hospitals, concur in strongly recommending: the warm-bath, with strong frictions of the surface of the body; and then a draught of some warm fluid, generally gruel, was given. In some instances, when the exhaustion was great, and the circulation not easily restored, a little wine was added to the gruel; "but this was done only when extreme necessity demanded it, and with great caution and reluctance." The head was shaved. When there was strength enough remaining to sustain without peril the action of vomiting, a gentle emetic was administered immediately after the warm-bath. By these means the hot stage was generally brought on, often in a mitigated form. In a few cases the emetic cut short the complaint. In this stage of the disease, and in such patients, the propriety of the use of emetics will hardly admit of dispute; but, in the cerebral or gastric forms of fever, when re-action is about to take place, or has come on, especially in more vigorous subjects, emetics are hazardous remedies. The late Dr. HARVEY, of Dublin, indeed, hardly ever employed them in the form of fever just mentioned, in the paupers of Dublin; and his success in the treatment of fever at Stevens's Hospital has not been excelled, under similar circumstances. He was led to refrain from the use of them by observation of their ill effects; and it must add no small degree of authority to his opinion to state, that he treated typhous fever in the way in which it is now treated by the most judicious physicians, at a time when bark and wine were the means employed by all but a few physicians, who, like himself, were original observers, and who made judicious theory the guide of their practice. Theory, however, as well as experience, is in favour of the use of this remedy in the first stage of fever, before irritation of the stomach has acquired much intensity; and the suddenness with which it removes fever in this stage, when the morbid association of other organs has not been well established, lends no small degree of support to the doctrine of the gastric origin of fever. The disorder is attacked in its source by the emetic, the morbid action overcome by its agency, and, consequently, the sympathetic phenomena immediately disappear.

Nothing in the animal functions is more clearly intelligible to the physiologist than this circumstance; but, without the light of physiology, it is obscure, mysterious, and calculated to excite *surprise*, as it did in the mind of SYDENHAM. "When I have happened," he says, "to examine carefully the matter thrown up by vomiting, and found it neither considerable in bulk, nor of remarkable bad quality, I have been surprised how it should happen that the patient had been so much relieved thereby; for, as soon as the operation was over, the severe symptoms, namely, the nausea, anxiety, restlessness, deep sighing, blackness of the tongue, &c. usually abated and went off, so as to leave the remainder of the disease tolerable."

When re-action had commenced, that is, perhaps, when the morbid irritation has been communicated to the nervous system generally, and to the heart and arteries, bleeding from the arm, carried to such an extent as to produce nearly syncope, when no particular circumstances contraindicated it, was commonly resorted to by the author of this Report. The quantity of blood drawn varied from ten to sixteen or twenty ounces. Sleep very frequently followed, and a partial, or sometimes a complete, remission of the symptoms. In many instances the patient was found to have experienced only a partial or temporary relief from the first bleeding; in such instances it was repeated, and to the third or fourth time, from the arm or the temporal artery; and when cephalic symptoms continued, after what was judged to be a sufficient employment of general bleeding, leeches were applied to the temples. This, with the other common measures, and especially purgatives, were usually successful in the treatment of the "simple and cephalic forms of fever." The purgatives employed by the author were calomel with jalap, or the compound extract of colocynth, followed by a draught of infusion of senna with sulphate of magnesia. As some degree of gastric irritation is probably always present in fever, the purgatives above mentioned are not perhaps the most proper for general use. The mixture of tartar-emetic and sulphate of magnesia, as recommended by Dr. Cheyne, and which was first, we believe, generally employed by Dr. Harvey, is, most probably, preferable. The author, however, employed purgatives of the same kind, even when gastric inflammation was evidently present. After bleeding, and blisters, "when pain seemed to have taken permanent hold of one particular spot," and effervescing draughts, with sometimes a few drops of the tincture of opium, were employed, to diminish the irritation of the stomach: but, he says, "a more effectual relief was generally obtained by a few doses of calomel, containing five or six grains, in pills, combined with some of the resinous cathartics, or followed by a dose of oleum Ricini, or the infusion of senna with sulphate of magnesia, and repeated at the interval of six or eight hours."

The rest of the treatment in these cases comprises nothing peculiar, except in the use of blood-letting to a considerable extent, when the disease had run on to the third or fourth week without abstraction of blood or other necessary evacuations; and "more especially if, as it often happened, the patients had been confined to a close and foul apartment, and improperly stimulated, were generally found to display typhoid symptoms, or to have become cases of typhus gravior." Individuals under these circumstances were often brought into the hospital in a state of stupor of delirium, their eyes suffused, their tongues covered with a dry and dark-brown crust, and their teeth with sordes; their skin hot and parched, and beset with petechiæ. The warm-bath, strong friction of the surface of the body, and afterwards cold or tepid ablution, were first employed, and much improved the state of the patient.

"Whenever there was delirium, or any considerable tendency to it, or to other affections of the head, the abstraction of blood was ordered, provided that the state of the circulation, particularly the degree of pulsation in the carotid and temporal arteries, was such as to authorize this measure; and, if the action of these vessels was strong, a feeble pulse at the wrist was not considered a reason for abstaining from venesection. The blood was drawn from the temporal artery, or from a vein in the arm, and the quantity was determined by the previous circumstances, and by the effect produced on the pulse. The benefit that resulted from such an evacuation often exceeded the most sanguine hopes. In the course of twenty-four hours, the patient was sometimes found to have passed from a complete typhomania, and an almost total unconsciousness of external impressions, to a tolerably perfect possession of his faculties.

"After one bleeding, under circumstances such as I have described, to the amount of twelve or fourteen ounces, or in some severe cases to a larger quantity, the state of the patient was generally found, in the course of ten or twelve hours, to be materially improved. Yet the pulse was very often increased in fulness and strength, the skin was extremely hot, or disposed to become so, with a general increase of what is termed re-action; the delirium continuing, though generally in a degree more or less mitigated. In this state of things, the bleeding was generally repeated, and the quantity taken was often greater than at first; while the effect of its abstraction, in reducing the pulse at the time, was less considerable in proportion. The subsequent proceeding was governed by the result of these measures. If the same indications existed, a third venesection was ordered. It generally happened that two or three bleedings were sufficient to reduce the circulation through the brain within limits compatible with the safety of its functions; but cases have occurred, in which four, or even five, bleedings were adopted with manifest necessity, and with the effect of restoring the patient, and putting him in a state approaching to convalescence. When the symptoms of severe disease were not subdued by these means, under such circumstances as I have indicated, it was almost uniformly proved, by examination after death, that the brain had suffered irreparable injury."

Leeches were employed instead of venesection in very debilitated and exhausted subjects; with the ordinary auxiliaries, as blisters, purgatives, and subsequently more gentle laxatives, and the continued use of calomel.

The author's observations on the effects of calomel when productive of ptyalism, nearly accord with those of Dr. GRATTAN. When the influence of the mercury on the secretory organs had taken place, there was a gradual remission of all the symptoms of fever; and, he says, "if this favourable alteration did not happen when the signs of ptyalism came on, it was generally evinced, in the sequel, that some severe and irremediable disease had taken place in the lungs or the viscera of the abdomen." When the calomel produced too much purging, it was sometimes joined with opium. The ordinary prescription was, five grains of calomel and the same quantity of Dover's powder, every sixth hour. The addition of opium was very beneficial in cases of great general irritability, attended with preternatural acuteness of sensation, such as the long want of sleep is apt to induce. This remedy (the calomel) was used in all the forms of fever, after blood-letting, with the ordinary auxiliary remedies appropriate to the particular indications in different cases.

Wine was but rarely and sparingly employed. "It seemed to be requisite," the author says, "in some of those cases which were brought into St. Peter's Hospital, in the last stage of exhaustion and starvation, when a sinking pulse and cold extremities indicated that the powers of life were already sinking; but in such instances it was given diluted, and in small quantities. I believe it may be stated, that the cases in which it was given were, with very few exceptions, those which are marked in the Reports as terminating in death."

The practice in respect to food, ventilation, &c. was similar to that generally adopted in fever hospitals. It was made a rule, in regard to the diet of convalescents, that they should solicit the common house-diet at least a week before it was allowed them; and, when this was strictly observed, relapses but seldom occurred.

The author next proceeds to treat "of the causes of fever; contagion; spontaneous origin; circumstances which occasion it to become epidemical." On each of these points his opinions coincide with those of nearly all the physicians who have witnessed the progress of the late epidemy in Great Britain. Still he, with reason, thinks it proper that he should give his testimony on those points, and relate the principal facts on which his opinions are founded. We may just state, that he considers that the disease was propagated by contagion; and that he is disposed to believe that fever may originate from other causes than animal contagion, and then, under certain circumstances, be communicated from individuals thus affected to others by contagious influence. He thinks there is no other supposition that can explain the origin of that which has recently been epidemical, except the hypothesis of SYDENHAM,—the existence of a pestilential constitution of the atmosphere; an hypothesis which has the support of the opinion of Dr. JACKSON, and of many other physicians, who, like him, have observed the first appearance of epidemies of this kind under multitudes of different circumstances of climate, soil, social intercourse, &c. where neither contagion, *fomes*, nor marsh-miasmata, can satisfactorily explain their origin.

The author concludes this work with advancing some suggestions respecting the measures which might be beneficially employed to prevent the future diffusion of contagious fever through the city of Bristol; but as these, as far as original suggestions are concerned, relate merely to local circumstances, we need not enter into the particular consideration of them.

---

*Practical Essays on Strictures of the Urethra and Diseases of the Testicles; including Observations on Fistula in Perinæo and Hydrocele. Illustrated by numerous Cases, and an Engraving; and prefaced with some Remarks on Life and Organization.* By ROBERT BINGHAM, Member of the Royal College of Surgeons. 8vo. pp. 357. Longman and Co. 1820.

THE author's intentions in publishing this work, as it appears from the Preface, are to make known some cases of stricture which he considers to be particularly interesting, with his mode of treating them; to inculcate the importance of attending to the state of the general system, in the cure of local diseases; to develop a principle which he thinks is indisputable, that "all local disease commences in morbid nervous action;" to show the necessity of attending to the state of other co-existent disorders, in the management of any certain one; and to state the value of the several modes of treating stricture in particular cases, without that undue bias which the original discoverers of certain remedies have for the exclusive use of them. Besides this, he adds, "it is believed that many things have been placed in a clearer point of view than has hitherto been done. It is also thought that many parts lay claim to originality, and originality of that kind to which utility is inseparably attached." As all the propositions above stated, except that which attributes "all local disease to morbid nervous action," have long been truisms with well-informed surgeons, we shall endeavour to develop, as we proceed in a particular review of the work, whatever bears the semblance of originality; whilst we pass over in a more rapid manner that which is merely common-place, or similar to what must have been observed and thought by every experienced practitioner of common sense and intelligence.

We have not yet noticed the whole of what is contained in the Preface: the author here says, that he could not treat of the diseases which are the subject of this work, without going so far back into the analysis of vital actions "as to occasion a brief enquiry into life, its connexion with organization, and their mutual influence upon each other." Let this notion be but generally acknowledged as right, and be conformed to, and no one in future can write a book on medicine without prefacing it with an enquiry into "life, and its connexion with organization;" for stricture of the urethra is not better entitled to such a prelude, than any other disease of the human body.

The author's object is to prove, that life cannot be the result of organization. It is mere vanity to treat on this point, on the present occasion; for the knowledge of the fact, taking its truth for granted, does not tend to elucidate the nature of stricture, any more than it does to explain that of the colic, Saint Vitus's dance, the jaundice, or any other malady. Had the author traced up the principle to any law in pathology at all related to the subject of his work, the propriety of the conduct alluded to could not have been disputed; but this he has not attempted to do. Still, as the subject is one which has occupied inquisitive men in all ages, and is yet involved in much obscurity, the making the preface to a work on Stricture the medium for elucidating it might be excused, had the writer stated any thing new. But this he has not done: what he produces had been already said, and is familiar to all those who pretend to any depth of information on the subject. It relates, besides, to merely superficial views, and is not at all calculated to explain the more profound difficulties which involve this matter. To be convinced of this, he need only peruse the second book of the "Indications" of Daniel Pring.

We should not have dwelt so long on this subject, had not the author made a notice of it form part of the title-page of his book. Mr. Bingham may think our remarks severe; and it is probable that we are not in our kindest humour on this occasion. The reason may be, that we have been disgusted with the impertinent manner in which some of our profession have taken up this subject of late, and the unworthy motives which have evidently led them to consider it. Men well versed in philosophy have relinquished it, except in treatises *ex professo*; and now we find writers on diseases introduce it amongst subjects which are connected with it only in the remotest manner, to make an outcry against those who think proper to discourse on it with logical severity; whilst they themselves prate about it in a way that would be ridiculous, if the subject were not of too serious a kind to be thus trifled with, and were not the character of our faculty, also, materially injured by the conduct of these medical puritans.

As we have thus, in spite of us, been obliged to notice these polemical disputations, we shall add a few words, in order to explain the grounds on which the chief of them have rested: such an explanation may be useful to some few of our readers.

It is probable, and as nearly proved as any thing can be by negative arguments, that there exists in nature some influence, or power, which, acting on certain species of matter, produces certain modes of organization qualified to perform certain functions, the sum of which functions in any individual being is the life of that individual; at least, as far as life is engaged in the phenomena which become the subject of physiological consideration. This is the way in which the matter is regarded, and in which the term life is commonly used by physiologists; and, according to this view, life is the result of organization. There are some persons who choose to apply the term life to the supposed principle which is the cause of organization; and no one would wish to interfere with their barren speculations and vague discourses, if they did not intrude them where they should not appear; and, either from ignorance or folly, or from both, oppose them to their own misinterpretations of the opinions of others, for purposes which their rashness deprives them of the power of concealing. One of these persons has lately said, that "the man who attempts to inculcate such sentiments, (as that life is the result of organization,) must be actuated by a sense of innate depravity, and, probably, by remorse of conscience for crimes committed!" Men who talk thus will dispute for ever: the only way of acting towards them is to treat them with contempt, whilst they do not interfere with the conduct and pursuits of wiser men; but, when they do this, to use no delicacy in the chastisement of them: for these gentlemen are so cased in vanity and satisfaction, that they cannot feel gentle hits; one must take up a shilelah to them.

The first section of the work before us commences with a description of the various kinds of stricture which occur in the urethra. Adopting the notions most commonly admitted, the author says,

> "Three varieties of stricture can be distinguished in practice: the spasmodic, which can be dilated to the full size of the urethra; the permanent, which admits no dilatation; and the mixed kind, which partakes of the nature of both."

As this division is not peculiar to the author, but, on the contrary, commonly admitted by surgeons, it is not right to put it to the test of minute critical examination on the present occasion; but, we should remark, the propriety of it is very doubtful: it rests on the supposition that the membrane of the urethra is muscular, which is very far from being proved. All we know of it is, that it is an elastic mucous membrane, surrounded by extremely vascular parts, which are themselves enveloped by an elastic tissue; and, consequently, that the calibre of its canal is liable to be lessened whenever the surrounding parts are inordinately distended with blood, or when the elasticity of its own texture is more or less destroyed. Certainly, the very small extent of some species of stricture, especially that which impresses a bougie with a mark like what would arise from a piece of fine twine being tied around it, favours the notion that the urethra possesses circular fibres capable of positive active contraction, but by no means proves it.

After a description of the common forms of stricture, the author, in the second section, treats of "the theory and causes of stricture." His explanation of the origin of the first species of stricture rests on the supposition that the urethra is muscular: he says,

"It is well known that nervous irritation in muscular parts often occasions partial and obstinate contractions; and, when this effect takes place in the urethra, I believe it constitutes what is generally understood by the term spasmodic stricture; but, as this expression is also applied to any kind of stricture which can be dilated to the full size of the urethra, so nervous irritation, muscular contraction, and a certain degree of inflammation, may all be present in spasmodic stricture."

The inflammation is, probably, all that is necessary to take into consideration, in order to explain the nature of this form of the disease: the other opinion must, at least, be considered as hypothetical, until we are better assured that there are muscular fibres in the urethra.

A permanent stricture, the author thinks, is not always attended with alteration of the structure of the part; for he "has examined some preparations, in which the contraction appeared of a closer and more compact texture, simply because they occupied less space than when they were in a healthy state." The explanation which offered itself to his mind was, "that the parts had been held in a contracted state until grown rigid and unyielding." Or the parts, he adds, may have been thickened from effused coagulable lymph, which may have afterwards become absorbed, leaving the original parts "perfect as in health, only in a contracted unyielding state, because they were then united, and could only move together; whereas they were previously loose, and moved freely on each other." This is generally, we think, regarded as alteration of structure; but the author chooses to confine the term to a state in which "all traces of the former arrangement are effaced." Such a complete change as that just mentioned does take place in some cases of stricture, the part assuming a texture nearly resembling tendon or cartilage.

The author notices the origin of stricture, from a portion of the urethra having been destroyed by ulceration, and cicatrized so as to contract the diameter of the canal. He then considers the causes of strictures in general. Gonorrhœa, he properly considers, tends to produce them only by means of the irritation and inflammation which accompany that affection. Irritating injections and improper use of bougies, act in the same way. But the most frequent cause, he considers, is an abuse of the functions of the part, especially in the venereal act. It must be known to every surgeon how much the account given by Sir Everard Home of the structure and functions of the urethra, and the situation in which the first stricture ordinarily takes place, tends to substantiate this opinion. We are somewhat surprised that the author has not referred this opinion to its proper author, and noticed the arguments in its favour above alluded to.

Disorder of the digestive organs is regarded by the author "as a very common cause of stricture." The truth of this notion is by no means well established. Co-existence of diseases, or their consequent appearance and disappearance, does not prove their relation as cause and effect. They may both have a common origin in some more general derangement of the system. The author himself indeed remarks, in another place, that he has "met with a few cases of stricture, in which there was not the slightest evidence of disorder of the digestive organs; but I have never yet seen a single instance of stricture in the urethra combined with disorder of the digestive organs, in which there has not been very great reason to doubt, whether the stricture gave rise to the disorder in the digestive organs, or was itself a consequence of that disorder." "A predisposition to the disease," says the author, "may have existed in the constitution, and only have needed something to bring it into action. This is evinced in many patients by a contracted state of the foreskin; and, unless it previously existed, I conceive that many of the above-mentioned causes would be found inadequate to the production of stricture."

It will be necessary, probably, to admit the existence of such a predisposition in many cases; or, to speak more correctly, of a peculiar degree of disposition to it in some persons, (for every person must be predisposed to every disease with which he may become affected, or he would not have suffered it,) or such persons would not have stricture produced by causes which do not produce it in persons in general. "Diseases of the prostate gland," the author concludes with remarking, "kidneys, or any of the chylopoietic viscera, numerous as these diseases are, may each one of them prove the exciting cause of stricture in the urethra." A co-existence of such diseases is certainly not of rare occurrence.

In the third section, the author treats of "the symptoms of stricture." His account of them is concise, but sufficiently explicit.

The fourth section is appropriated to some "preliminary observations on the treatment of strictures;" and comprises an application of the foregoing principles respecting the causes of the disease to the means qualified to avert or alleviate it.

The next section relates to bougies; and contains a description of the properties and qualities of those in common use.

Some rather good remarks "on the mode of introducing bougies," constitute the next section; after which, the author treats "on the cure of strictures by the simple bougie."

The curative intentions in stricture, and the agency of the common bougies, are first considered. The worst of those cases, which will scarcely bear to be touched with a bougie without being aggravated, the author says, "are usually accompanied with some disorder of the general health and peculiarity of constitution;" and it will be advisable, he adds, to use no bougie until the general health be improved, "or the irritability of the constitution and of the part be somewhat allayed."

Explicit directions follow, on the mode of using the common bougie; and some proper remarks on the impropriety of too frequent introduction of the instrument when it produces much irritation, and on the management of several symptoms of frequent occurrence when it is employed.

The eighth section is "on the cure of strictures by argentum nitratum." The author's observations on this subject are judicious, but they are not sufficiently comprehensive, to supply the place of those of the surgeon who has brought the practice into common use. Almost every man who has made an important discovery, or developed the application of it, has an undue bias for its use; and such is undoubtedly the case with regard to the surgeon here alluded to, in the employment of caustic in the treatment of stricture; but it requires nothing more than common sense and a little careful observation, to make a distinction between the dictates of enthusiasm and prudence; and, with this, his observations on this remedy must prove of essential service to the surgical practitioner. We should have noticed Sir Everard Home's Treatise on Strictures in a more particular manner than Mr. Bingham has done, had we written, *ex professo*, on the use of the lunar caustic in treating these affections.

When the cauterizing plan is determined on, the author thinks the following is the best mode of applying it:

"A flexible gum cannula, as large as can be passed down to the stricture, should have a bougie introduced along it till its point reaches beyond the end of the cannula. The mere point of the bougie may then be a little softened by heat, and pressed on some very finely-levigated argentum nitratum till its surface is well covered with it, and then drawn back within the cannula a little way. The cannula, containing the armed bougie, ought to possess a proper curvature, according to the situation of the stricture; and, having some unguentum cetacei smeared upon it, should be passed down to the stricture, where it is to be held, whilst the bougie is urged forward till its point comes in contact with the part on which it is designed to operate; against this the bougie is to be pressed for a few seconds; and then, being once more drawn within the cannula, they are both together to be removed from the urethra."

When a bougie can be passed through the stricture, the author recommends that the caustic should be used in the form of an ointment, composed of from two to eight grains of the caustic to half a drachm of lard. His mode of using it is as follows:

"Select a flexible gum cannula of the full size that can be passed down to the stricture, introduce some of the caustic ointment within the point of the cannula; then, having anointed the external surface, pass it down to the stricture; a bougie is to be introduced along the cannula, and passed gently through the stricture: it may be allowed to remain there a short time, and then be drawn back again into the cannula. It may be re-passed gently through the stricture once or twice more; and then, the point of the bougie being drawn within the cannula, both may be taken from the urethra."

Another mode he proposes for using this ointment, is to put it into a groove made near the point of a wax bougie. This bougie is to be passed, either simply or in a cannula, through the stricture, so that the ointment may be applied to the diseased part.

We do not think that either of the above measures is likely to supersede, in the practice of other surgeons, the common armed bougie in general use. It does not strike us that the caustic could be applied with equal accuracy by the former measures, and certainly not with less danger of doing unnecessary injury. The author attributes the introduction of the use of caustic, in ointment or in powder, on the extremity of the bougie, to Mr. HOWARD; but he will find, on looking into ALPHONSO FERRI's treatise *De Caruncula*, published in 1650, that the practice is of much older date. Those who may be disposed to adopt this method of using caustic, may, perhaps, think some of the other applications of Ferri worthy of trial; as verdegris, orpiment, red precipitate, &c.

The ninth section is "on the cure of strictures by kali purum." The author commences with some general observations a little unfavourable to the use of this remedy, and a citation of Mr. WHATELY's directions for employing it: he then makes some remarks on its mode of action, especially on Mr. Whately's observation, that it "abrades the membrane of the stricture without producing a slough;" which account of its agency the author objects to. He says, "it appears to me, however, that this explanation is not correct; for, if the surface of the stricture were abraded, or rubbed off, as the term implies, it must lay open vessels that would bleed, if no slough were formed and remained attached to close their apertures." We conceive that Mr. Bingham has not been in the habit of seeing the kali used as a caustic, or he must have observed, what we believe Mr. Whately intends to signify, that the kali causes a decomposition of the surface to which it is applied without producing an adherent slough; and he would have found that the part thus decomposed was separated without any hemorrhage taking place from the surface beneath. Mr. Bingham thinks that "the circumstance which most of all encourages the idea that some part of the stricture is abraded by the use of the kali is, that, immediately after its use, a larger bougie can be passed through the stricture than would have been previously made to enter." This he explains by the secretion produced by the kali relieving the turgid vessels of the inflamed part, by its removal of the "spasm" of the stricture, and by the saponaceous fluid it causes to be formed in the urethra. He is "confirmed in the truth of this mode of explaining the effects of the kali purum upon stricture, by experiencing precisely the same results from the application of the potassæ subcarbonas, the natron preparatum, or even Windsor soap."

The two following sections are appropriated to the author's observations on the cure of strictures by the subcarbonates of potash and soda. His object is to show that these medicines will produce all the good that can be derived from the pure kali, without the danger attendant on the use of that caustic preparation. The author relates several cases, with the view of proving the truth of this statement; but they do not even convince us that these medicines possess any considerable degree of merit, much less that they can supersede the caustic kali. That the subcarbonate is a "milder medicine," is true; but the causticity of the pure kali is a desirable quality on many occasions. There are, it is true, some cases of stricture, in which an irritating application greater than the simple bougie, and yet not escharotic, may be desirable; and then the subcarbonates may, perhaps, be useful remedies, as well as red precipitate, verdigris, and the various other things which have been favourite medicines with different surgeons.

The author applies the subcarbonates in several ways: first, inserted in the end of a wax bougie, in the same way as the pure potash is done in Mr. Whately's bougie; secondly, inserted in the end of a cannula, within a mass of lard, which is to be driven out by a bougie, in the same manner as the caustic ointment described on a former occasion; the third method is to pass the bougie, armed in the first way, through a cannula.

Another remedy, which the author thinks is a "useful auxiliary to the bougie," is the strong mercurial ointment of the Pharmacopœia. He says,

"Six cases have been perfectly cured by it in the course of my practice. To three of these other remedies had been applied without any good result, but immediately improvement followed the use of the unguentum hydrargyri fortius. In a seventh instance, in which all other means seemed to disagree, it proved beneficial, and the case ultimately did well; but I am inclined to think that the re-establishment of the patient's general health had much to do in the cure. Two more I lost sight of when they were very nearly well; and in a tenth case, now under treatment, and to which the unguentum hydrargyri fortius continues to be applied, the improvement is as great as the time and circumstances will admit. Hitherto I have not used the unguentum hydrargyri fortius to a single case of stricture in the urethra, when it has not proved beneficial; but this is not sufficient to justify an inference that it will never prove worse than useless."

Although but few surgeons in London can produce, from the practice of a few years, so many cases of stricture successfully treated, as the author has done in this book, and though the greater number of practitioners are far from finding this affection, in general, so perfectly tractable, yet they will not fail to infer that, in several of those related by the author in this section, the simple bougie would, in all probability, have been equally beneficial. It is not fair to argue that, because a man has not borne the introduction of a simple bougie several times without great inconvenience, and then at length, after the lapse of several days, has not felt much from one smeared with blue ointment, that the ointment has prevented the injurious effects of the bougie; neither is it a good argument, that the benefit which may have followed the use of such a bougie after this, is due to the ointment; as changes of this kind are common in the treatment of stricture by the simple bougie. We cannot believe that the blue ointment can have so much effect on strictures as the author imagines; though we know that it is thought beneficial, when used in the same way, in strictures of the rectum, by several surgeons: this preparation of mercury appears to be too inert to exert any considerable influence on a part like the urethra, to which it can be applied only in a very transient manner.

The author says, "sometimes it produces no sensation different from that which the bougie alone would excite; at other times, patients have told me it felt warm, and sometimes very warm, but never amounting to a painful sensation:" and he adds, in a note, that he has met with three patients in whom "it has occasioned a burning pain for some minutes after the bougie was withdrawn." This we should expect; not from the agency of the mercury, but from the *salt* contained in the lard with which the ointment is usually made; or, when the lard is deprived of its salt, from the *rancid* state of the ointment. We are disposed to believe that as much good would be produced by smearing a bougie with lard as with the blue ointment.

The author next treats on fistula in the perinæum, communicating with the urethra, in connexion with stricture. On this subject he advances nothing original of remarkable importance. We have only to say on this subject, that other surgeons have found this affection more difficult to remedy than the tenor of the author's observations would indicate; and we suspect that the imagination has directed his remarks on it more than practical experience.

The fifteenth section is on "false passages in the urethra," produced by the bougies or catheter. A description of their ordinary seats, and of the way in which they are produced, and some proper observations on the means of avoiding them in the introduction of a bougie, &c. are first given. For the treatment of them, the author recommends the practice of laying them open with the knife, when they obstruct the passage of instruments that are necessary for the treatment of stricture. This operation is no trifling matter, but it may certainly be necessary in some few cases.

The next section is "on diseases of the testicle." The author thinks, that the origin of chronic enlargement of the testicle from stricture, is not sufficiently known by surgeons, or not enough considered. "For," he says, "granting that few practitioners would see a chronic case of enlarged testicle, without ascertaining whether or not any stricture existed in the urethra, if they could not otherwise satisfactorily account for the disease, yet, when other causes were apparent, and those such as might be deemed equal to the production of the morbid state of the testes, as for instance when it seems to result from gonorrhœa; then, I believe, the examination of the urethra, by the introduction of a bougie, would very commonly be omitted." Here the author appears to us to resemble a little a physician we know, who, because he in two or three instances found disease of the heart, on dissection after death, where he had not expected it, can see now nothing but diseases of the heart in every disorder of the thoracic and abdominal organs; and he is so far infatuated on this subject, as to be past all hope of recovery from his *monomania*. If he opens a body, and finds disease of the heart, he has a long story to tell of all the things it had *simulated* within the last ten or twenty years. At one time it was inflammation of the liver; at another, irritation of the bladder or uterus; at another, ophthalmia or colic, and so on; every malady the patient had suffered was really nothing more than this disease of the heart wandering about the body in masquerade. If there are no signs of a morbid state of this organ where he had stated it to have existed, why then, it had been present, and had disappeared before death. So we think it is with our author, in some degree, in regard to stricture. Surely, when a surgeon finds another sufficient cause for disease of a testicle, he would not look for its origin in stricture of the urethra; though we have no doubt but that a surgeon whose imagination is haunted with the idea of stricture, might believe he found one as often as he sought for it on such an occasion.

The author next notices the origin of swelled testicle from gonorrhœa and other diseases of the urethra, and states it to be comparatively a rare occurrence: "we are then," he continues, "necessarily led to conclude, that some difference must exist between those cases which cause an affection of the testicle, and those which do not. Perhaps some cases may be explained by supposing that a stricture, or some other morbid affection, may have existed in the urethra prior to the gonorrhœa, but that the additional irritation of the gonorrhœa was necessary to enable the stricture, or other morbid affection, to cause an enlargement of the testis; or, on the contrary, the gonorrhœa might have been incapable of exciting hernia humeralis, [humoralis,] without being accompanied with the additional irritation of the stricture, or other morbid affection." Such suppositions are not at all necessary on this occasion, in order to explain why inflammation of the testicle will arise from gonorrhœa in one case and not in another, any more than it is necessary to suppose that some disease of the spinal marrow existed previously to a wound which has produced tetanus, because tetanus does not follow every wound of a similar kind; and have therefore no claims whatever on our credit, without facts in support of them. The author relates three cases which appear to him to justify his opinion. In the first, five weeks after the appearance of the swelling of the testicle, which took place as a gonorrhœa was getting well, but which had in that period once nearly subsided, the author says he detected a stricture "in the bulbous part of the urethra." A stricture perhaps existed here, and it may have had something to do in the production of the swelling of the testicle; but we have doubts about it. A stricture "in the bulbous part of the urethra" is of very rare occurrence, we believe, and we ourselves never met with one instance of it; and we know that the natural contraction of the urethra behind the bulb has often been mistaken for stricture. In the second case, it appears more probable that stricture did exist, and that it had some influence in producing the disease of the testicle; though the account of it given by the author is not very convincing in respect to either of those points.

Some cases of hernia humoralis follow, "which seemed," as the author says, "to be brought on entirely by an inflamed or irritable state of the posterior part of the urethra." Although cases of this kind are of frequent occurrence, those here alluded to will be perused with a considerable degree of interest, from their presenting some peculiar circumstances worthy of attention. These cases lead the author to make some good remarks on the importance of directing our chief attention to the removal of the disease of the urethra in cases of this kind. He introduces an account of the mode of treating swelled testicle attending gonorrhœa adopted by Mr. Ridgway, surgeon to the Rifle Corps. The remedy is an injection of a solution of nitrate of silver, in the proportion of from ten grains to two scruples of the salt to an ounce of distilled water. It is to be used with an ivory syringe night and morning, and repeated each time until "the membranous lining of the urethra appears white or sloughy; which may be ascertained by looking between the lips of the external orifice. The stimulus of the injection at first excites a copious pouring-out of the mucus of the urethra, which coagulates on meeting with the injection: all this coagulated matter must be pressed out of the urethra as often as it forms, and the injection must be used again and again till it produces no more coagulated mucus; for, without this is done, the injection cannot have free access to the surface of the urethra, and then it cannot be expected to operate properly." This practice appears to have been particularly successful, and was used whether or not the discharge from the urethra had been suppressed.

In some cases it is said to have simultaneously removed the discharge and the disease of the testicle. Here we must suppose that the disease of the testicle was in *direct* sympathy with that of the urethra; whilst others, in which the discharge, the ardor urinæ, and tenderness about the urethra, cease on the coming-on of the hernia humoralis, and re-appear as this subsides, seem to be in *reverse* sympathy. This appears to be the only way of satisfactorily accounting for all the circumstances in these affections.

Except that the author does not appear to have regarded the possibility of the disease of the testicle acting in reverse sympathy with the urethra, his views of these affections are tolerably accurate. He says, the spontaneous re-appearance of the discharge from the urethra, when the swelling of the testicle subsides, is owing, he apprehends, "to the irritation in the urethra changing or lessening." He seems to forget that every symptom of irritation of the urethra has been absent in the interval, in the greater proportion of cases.

Amongst his cases of disease of the testicle, there is one in which the enlargement of the gland disappeared to a certain extent under the influence of mercury, when an attack of acute inflammation of the gland came on; and, after the subsidence of this inflammation, the testicle was reduced to its natural size. Here, the author says, "either the stimulus necessary to excite the absorbents into action unavoidably produced inflammation, or else the absorbents were excited into action by the inflammation, their action being the last which occurs in inflammation:" the latter inference he thinks, with apparent propriety, is most probable; and hence he suggests the practice, in some cases, "of rousing an enlarged testicle into a state of active inflammation, with a view to its subsequent reduction." This practice is, we think, hazardous in the extreme, and hardly warrantable in any instance, unless cases of the kind just alluded to were much more common than they are found to be, and destructive ulceration in consequence of inflammation coming on in such a state of the testicle, much less frequent than is presented in the results of the common course of practical observation. It is impossible to anticipate, with any approach towards certainty, what will be the consequence of active inflammation in a gland in a state of chronic enlargement; as fatal disease, of what is called a malignant or cancerous nature, has often ensued from such circumstances, even when the original affection appeared to be the result of external violence. The introduction of a bougie, rather larger than what the membranous and prostatic parts of the canal will admit with ease, is the mean the author proposes for exciting inflammation of the testicle.

The work concludes with some remarks on suspensory bandages for the testicles, and on the treatment of hydrocele. The common calico bandage he has known to produce uneasiness, by its friction, and by its want of elasticity, by which it might accommodate itself to the parts. He recommends in place of it a pair of leather drawers, which are kept up by spring braces, the buttons supporting which are placed wide apart in front, so that the line of draught extends down the sides of the abdomen nearly to the spines of the ilia, and thence obliquely in the direction of the groins to the perinæum. The author mentions having seen some instances where this mode of support was attended with remarkable advantages; and he advises its use also in variocele, and in hydrocele, with the view of exciting the absorbents, by its pressure, in the latter disease. The author seems to think that this measure, and the application of stimulating lotions to the scrotum, might remove hydrocele, in many of the cases which are ordinarily treated only by the palliative method of evacuating the fluid by the operation.

In passing through this work, we have had occasion to reprove some parts of it which we have noticed in a particular manner, and to speak in terms of approbation of many others; whilst we have met with but little that we consider deserves being censured with much severity. The degree of merit of the work as a whole would then have been already indicated, were it not that we have let but few of what we believe to be faults escape our reprehension; whilst, from the necessary conciseness of this article, we have been obliged to neglect speaking of much that is of more or less practical utility. To give the reader, then, a more correct idea of the judgment we have formed respecting it, we should add, that it comprises many accurate observations and judicious reflections of considerable practical value, and such as will be new to at least the younger members of the profession; to whom we may confidently recommend it, as calculated to be of much utility to them in practice, provided they make a distinction between the dictates arising from that partiality which all men have in favour of measures and opinions originating with themselves, and the precepts inculcated by cooler and more severe contemplation.

### CRITICAL ANALYSIS

OF

RECENT PUBLICATIONS, IN THE DIFFERENT BRANCHES OF MEDICINE AND SURGERY;

